# Supplementary figures and images for: Transition from a meiotic to a somatic-like DNA damage response during the pachytene stage in mouse meiosis
Source: PLoS Genet. 2019 Jan 22;15(1):e1007439. doi: 10.1371/journal.pgen.1007439 (PMC6358097; doi:10.1371/journal.pgen.1007439)

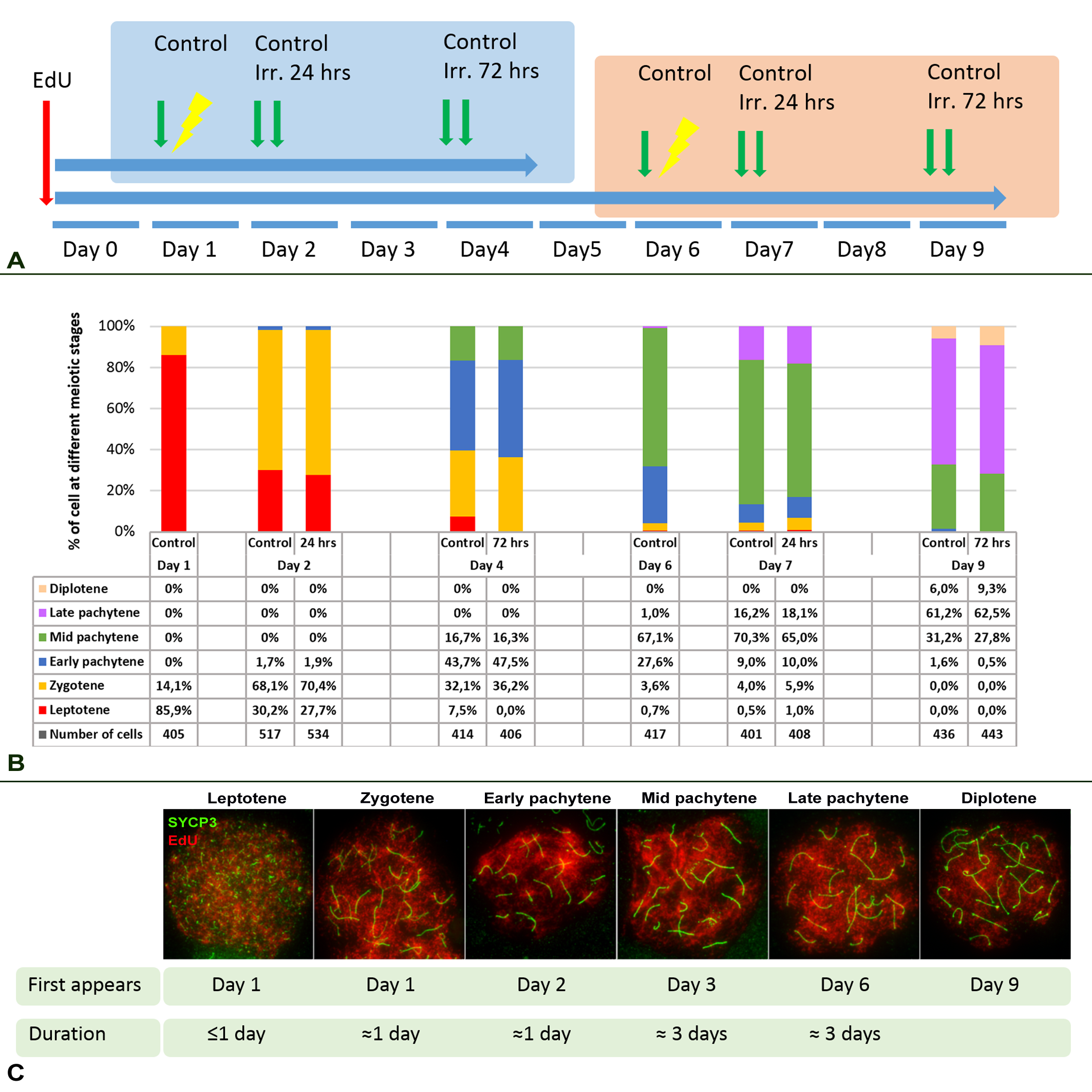

Supplement: S1 Fig — A) Schedule for EdU labeling and analysis. Mice were injected with EdU at day 0. Then, control samples were taken at day 1, day 2 and day 4. A subset of mice were irradiated and samples collected 24 (day 2) and 72 hours (day 4) later. In an additional experiment, control samples were taken at day 6, day 7 and day 9. A subset of mice were irradiated at day 6 and samples collected 24 (day 7) and 72 hours (day 9) later. B) Analysis of the proportion of spermatocytes labelled with EdU at different stages. Bars in the graph indicate the proportional contribution of each stage at the different time points analyzed. Percentages and the total number of cells analyzed are indicated in the table below. The results of day 2, day 4, day 7 and day 9 were compared between control and irradiated cells. Chi squared test showed no statistical differences in any of the days analyzed (X2 = 0.78, 2 degrees of freedom (df) for day 2; X2 = 8.01, 4 df for day 7; X2 = 6.92, 3 df for day 9), excepting day 4 (X2 = 32.08, 3 df). This is due to the conspicuous absence of leptotene cells 72 hours after irradiation. When this cell population was not considered, differences were not significant (X2 = 0.48, 2 df). C) EdU labeling (red) and SYCP3 staining (green) in spermatocytes and estimated length of meiotic stages. The duration of each stage was estimated on the basis of the day after EdU injection in which each stage is detected for the first time. The approximate duration of 1 day for leptotene, zygotene and early pachytene is consistent with previous reports [54,99]. (TIF) [file pgen.1007439.s002.tif]

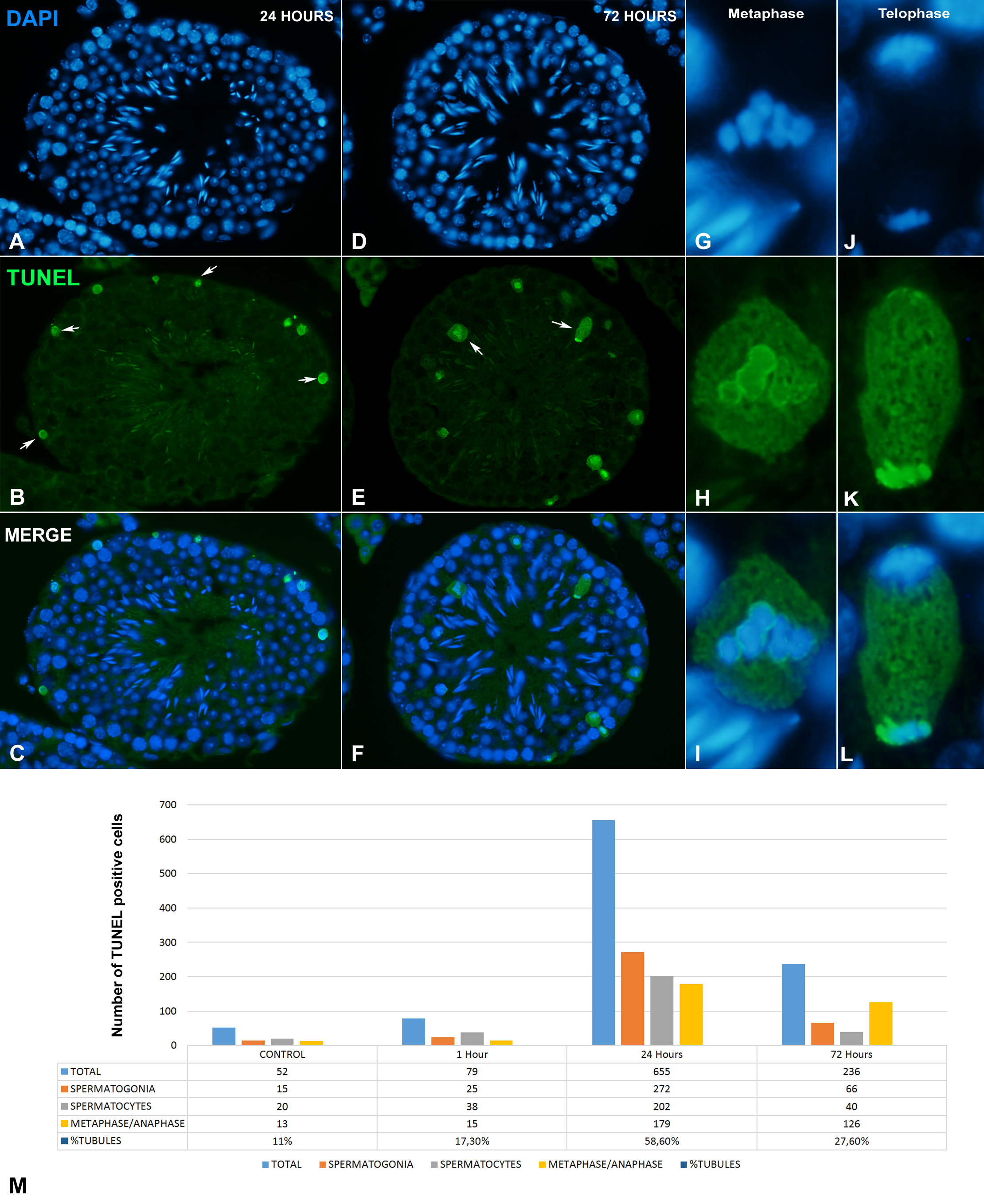

Supplement: S2 Fig — TUNEL (green) and DAPI (blue). (A-C) Section of a seminiferous tubule 24 hours after treatment. Apoptotic cells (arrows) are found close to the basal stratum of the seminiferous epithelium. (D-F) Section of a seminiferous tubule 72 hours after treatment showing apoptotic cells at metaphase or telophase (arrows). Enlarged images from D-F showing apoptotic cells at metaphase (G-I) and telophase (J-L). (M). Quantitative distribution of apoptotic cells. Total number of apoptotic cells were recorded in 300 seminiferous tubules. Peak apoptosis is observed 24 after irradiation with 58.6% of tubules showing at least one apoptotic cell. At this time, spermatogonia are the most affected population, followed by spermatocytes and cells undergoing division. After 72 hours of recovery, the total number of apoptotic cells decreases with only 27.6% of tubules showing apoptotic cells. At this recovery time, the majority of cells undergoing apoptosis are at metaphase or anaphase. (TIF) [file pgen.1007439.s003.tif]

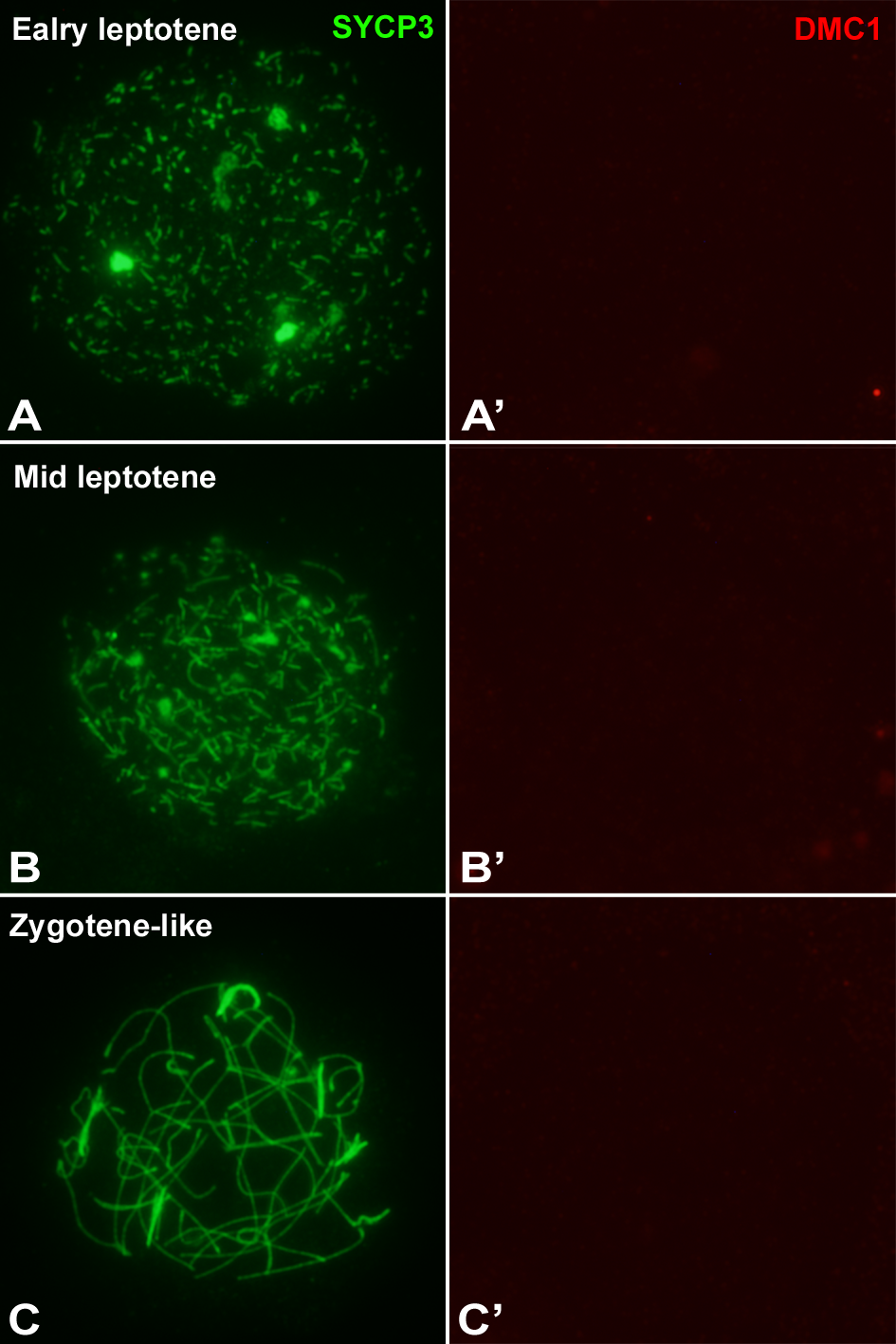

Supplement: S3 Fig — (A-C) SYCP3 (green) and (A’-C’) DMC1 (red) at early leptotene (A, A’), mid leptotene (B, B’) and zygotene-like (C, C’). No specific signal of DMC1 is detected at any of the meiotic stages in this knockout model. (TIF) [file pgen.1007439.s004.tif]

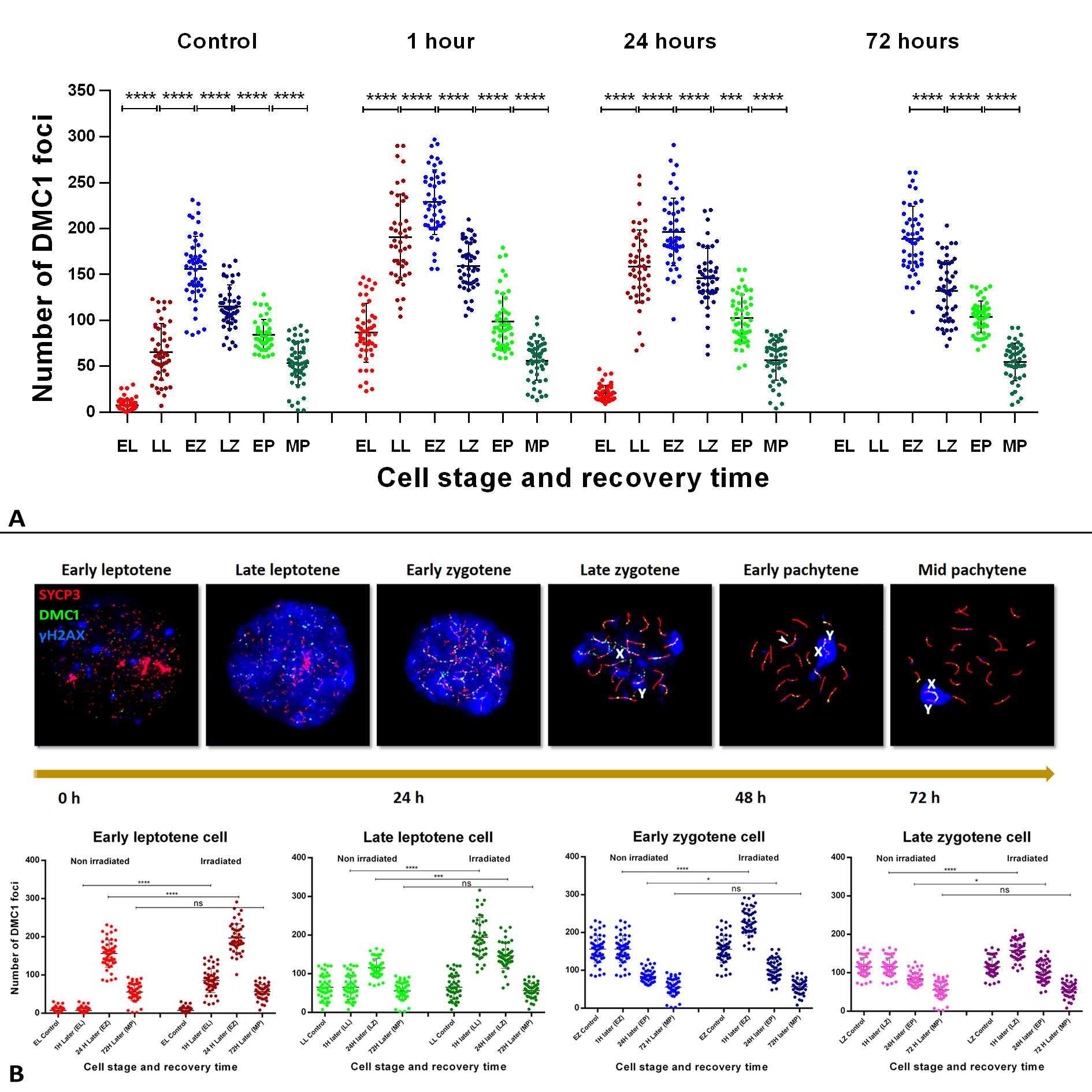

Supplement: S4 Fig — A) Analysis of DMC1 distribution by time of recovery. Six substages were considered (EL: early leptotene; LL: mid-late leptotene; EZ: early-mid zygotene; LZ: late zygotene; EP: early pachytene; MP: mid pachytene). The six populations, including early leptotene, are clearly distinguishable in the control. A low number of foci is found in EL cells but numbers increase in LL, peak in EZ and then gradually decrease in LZ, EP and MP cells. ANOVA analysis showed statistical differences (p≤0.0001) for the control and the three recovery times, and Tukey's multiple comparisons test for individual comparisons between different stages showed statistical differences in all cases (*: p≤0.05; **: p≤0.01; ***: p≤0.001; ****: p≤0.0001). B) Distribution of DMC1 foci arranged according to the recovery time after irradiation and the putative stages that cells should have reached at that time. We arranged four cell populations: early leptotene, mid-late leptotene, early-mid zygotene and late zygotene. For each case, irradiated cells were compared with their respective control counterparts and statistical differences indicated (ANOVA and Tukey's multiple comparisons test). All cells advanced to mid-pachytene after 72 hours of recovery. Since DMC1 is not inducible at this stage, all cells reached DMC1 control levels, regardless of whether repair had been completed or not. EL: early leptotene; LL: mid-late leptotene; EZ: early-mid zygotene; LZ: late zygotene; EP: early pachytene; MP: mid pachytene; ns: non-significant; *: p≤0.05; **: p≤0.01; ***: p≤0.001; ****: p≤0.0001. (TIF) [file pgen.1007439.s005.tif]

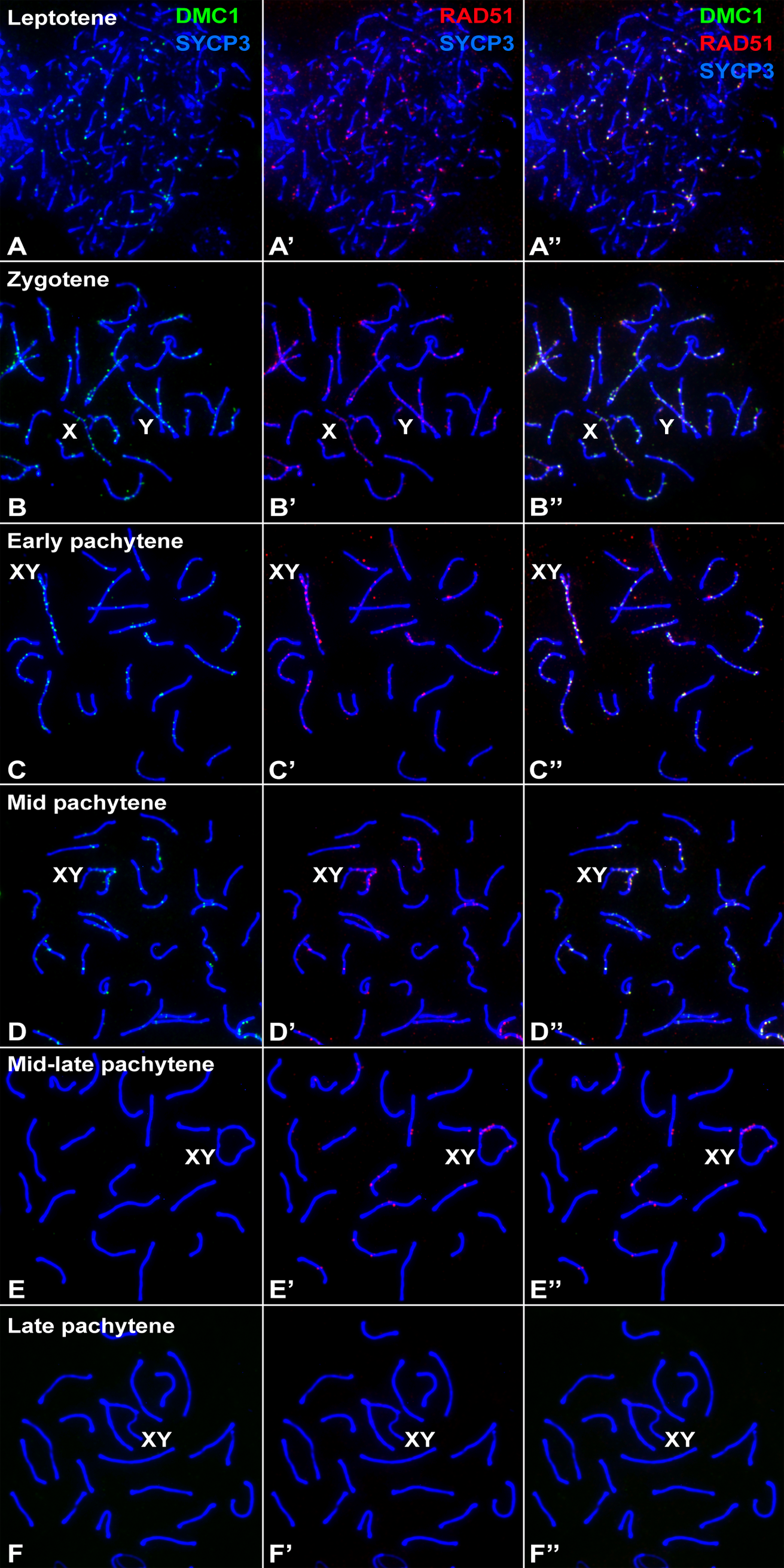

Supplement: S5 Fig — SYCP3 (blue), DMC1 (green) and RAD51 (red). Merge of the (A-F) SYCP3 and DMC1 channels, (A’-E’) SYCP3 and RAD51 channels and (A”-F”) SYCP3, DMC1 and RAD51 channels. DMC1 and RAD51 are largely co-localized in foci observed from early leptotene to mid-pachytene (A-D). However, DMC1 and RAD51 signal on these foci are usually not identical in size or shape. Moreover, there are some instances in which either of the two proteins seem to form single foci. At mid-late pachytene (E-E”), DMC1 is no longer present on the chromosomes, but RAD51 is still abundantly observed on both autosomes and sex chromosomes (X and Y). By late pachytene (F-F”), neither DMC1 nor RAD51 are observed. (TIF) [file pgen.1007439.s006.tif]

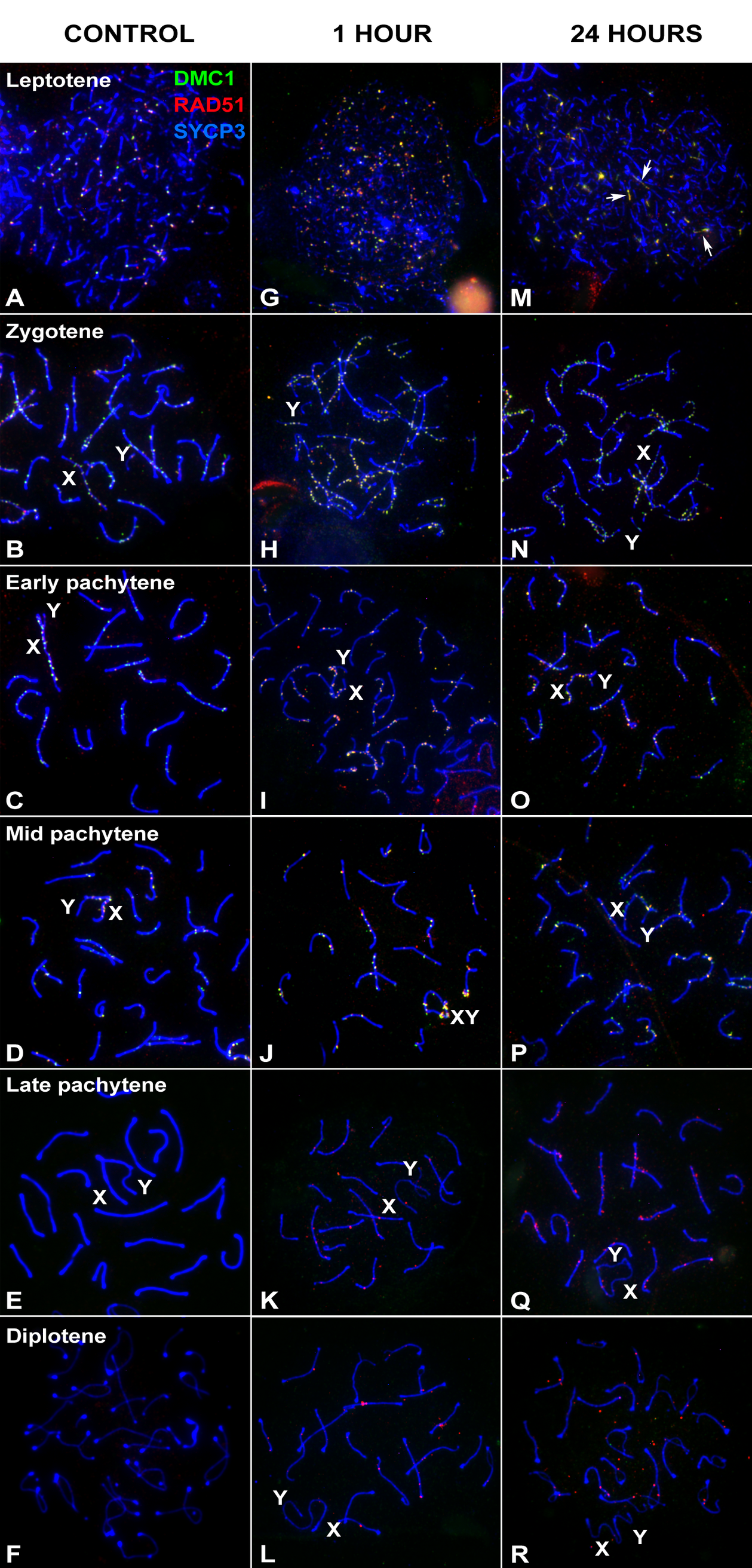

Supplement: S6 Fig — SYCP3 (blue), DMC1 (green) and RAD51 (red). (A-F) Control. The cells shown in A-D are the same as those shown in S5 Fig. (G-L) 1 hour after irradiation. As shown in Figs 4 and 6, both DMC1 and RAD51 become more abundant after irradiation with both proteins being present in the same foci in most cases; however, the overlap in signals is not identical in many instances as the sizes and shapes of foci of the individual proteins differ. From late pachytene onwards, only RAD51 foci are detectable. (M-R) An analogous result is found 24 after irradiation. Filaments containing both DMC1 and RAD are observed in some spermatocytes (arrows in M). (TIF) [file pgen.1007439.s007.tif]

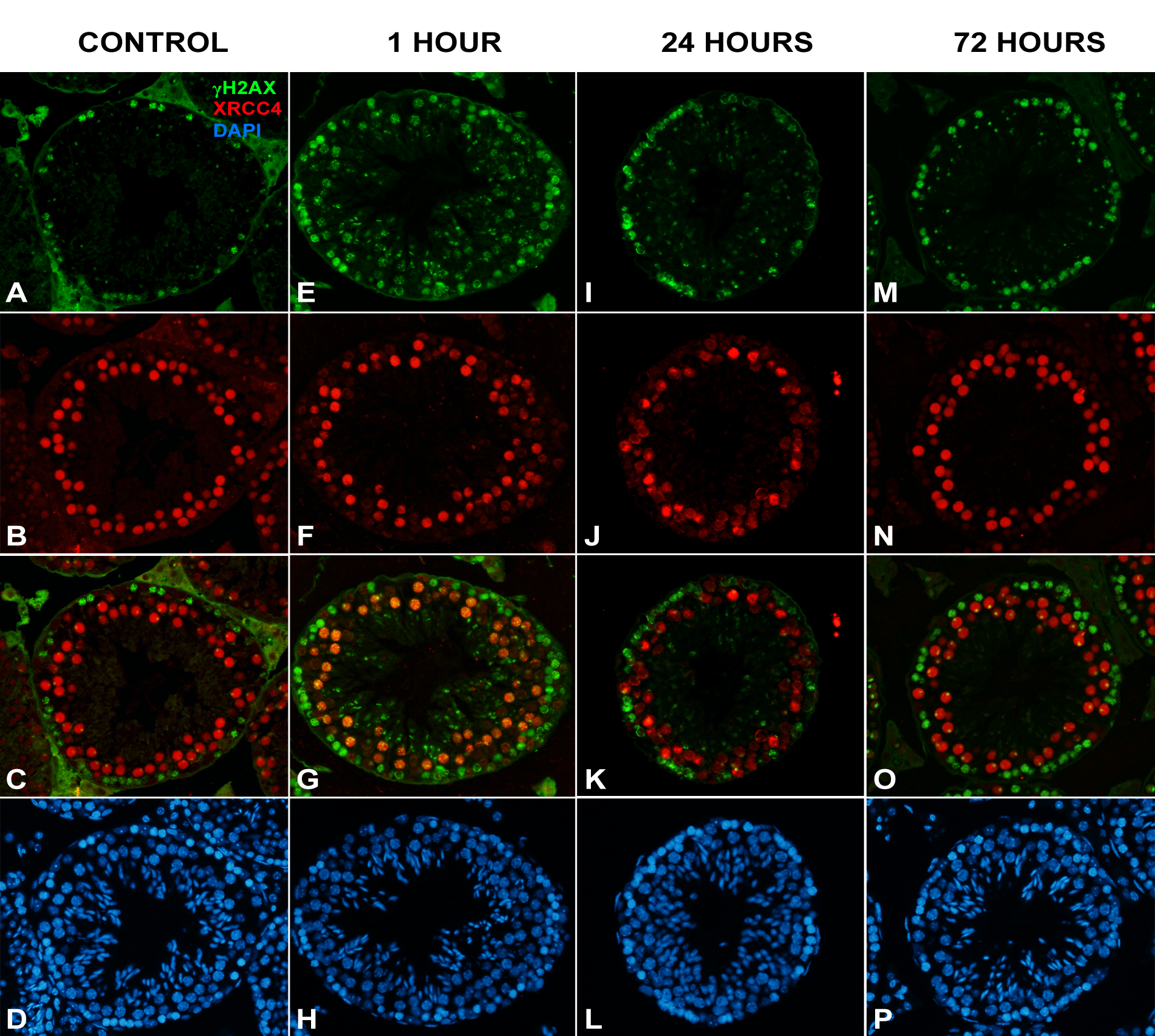

Supplement: S7 Fig — γH2AX (green), XRCC4 (red) and DAPI (blue) in seminiferous tubules at equivalent developmental stages. (A-D) Control. Basal layers of spermatocytes, corresponding to leptotene and zygotene, are broadly stained with γH2AX and devoid of XRCC4. Spermatocytes in the interstitial strata of the epithelium show an almost inverse labeling pattern, with abundant XRCC4 and nearly no γH2AX staining. (E-H). 1 hour after irradiation. Cells showing broad γH2AX labeling in the basal strata are again devoid of XRCC4. In contrast, spermatocytes stained with XRCC4 now also have an abundance of γH2AX localized foci, corresponding to the late γH2AX response. No noticeable increase in the intensity of XRCC4 labeling is observed. (I-L) 24 hours and (M-P) 72 hours after irradiation γH2AX tends to return to control levels. No variation of XRCC4 labeling is observed after longer periods of recovery. (TIF) [file pgen.1007439.s008.tif]

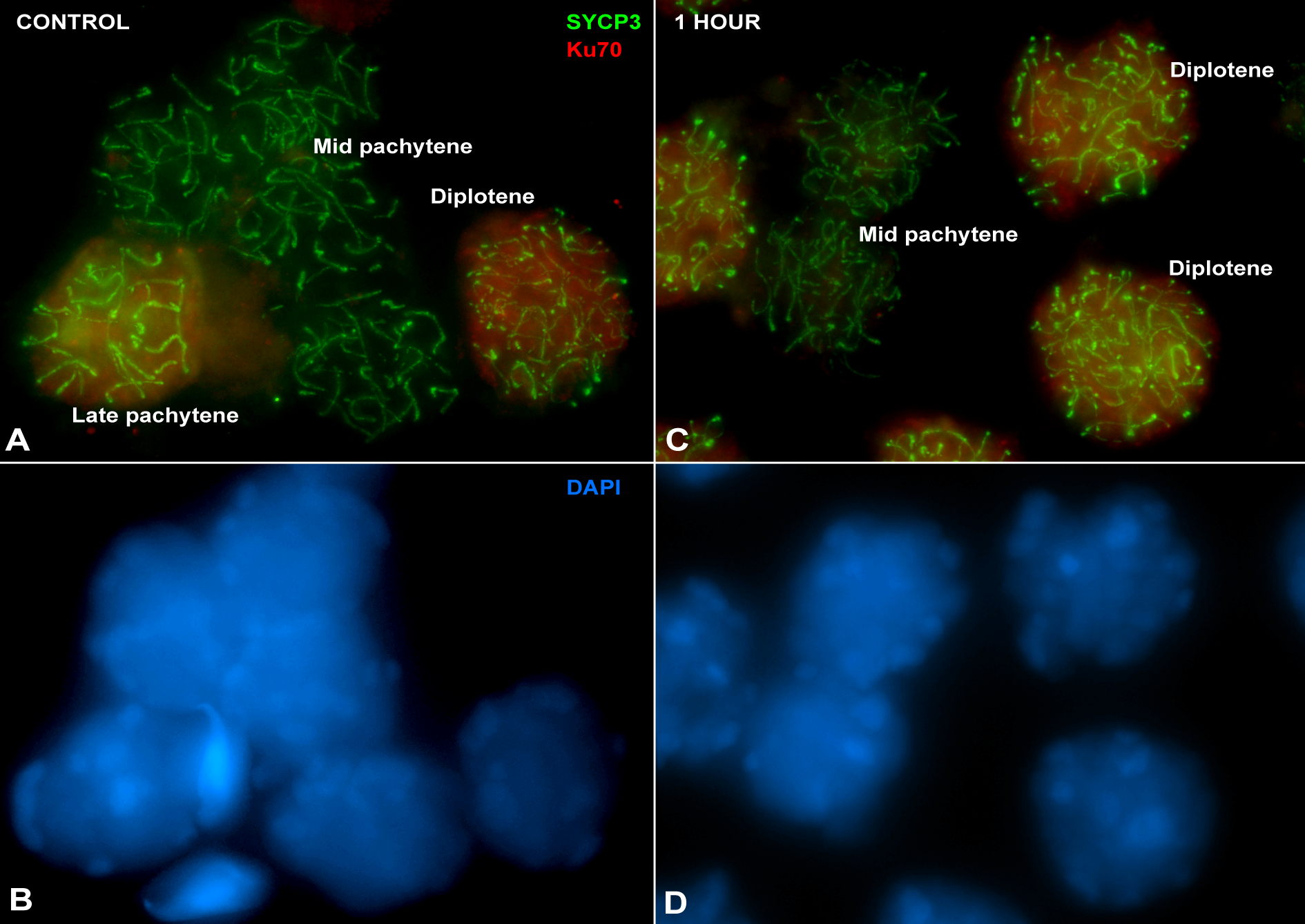

Supplement: S8 Fig — Ku70 (red), SYCP3 (green) and DAPI (blue) in squashed spermatocytes at different meiotic stages. Z-projections of image stacks across spermatocytes. Ku70 seems to be absent in early or mid-pachytene cells in both control (A-B) and irradiated spermatocytes (C-D). At late pachytene, a clear labeling is observed in the nucleus, covering most of the chromosomes. The labeling is more intense in diplotene spermatocytes. No evident differences in the intensity of labeling are observed between control and irradiated cells. (TIF) [file pgen.1007439.s009.tif]
